# Vps35 haploinsufficiency results in degenerative-like deficit in mouse retinal ganglion neurons and impairment of optic nerve injury-induced gliosis

**DOI:** 10.1186/1756-6606-7-10

**Published:** 2014-02-11

**Authors:** Wei Liu, Fu-Lei Tang, Joanna Erion, Hang Xiao, Jian Ye, Wen-Cheng Xiong

**Affiliations:** 1 Department of Ophthalmology, Institute of Surgery Research, Daping Hospital, Third Military Medical University, Chongqing, China; 2 Institute of Molecular Medicine & Genetics and Department of Neurology, Medical College of Georgia, Georgia Regents University, Augusta, GA 30912, USA; 3 Charlie Norwood VA Medical Center, Augusta, GA 30912, USA; 4 Department of Geriatrics, Southwest Hospital, Third Military Medical University, Chongqing, China

**Keywords:** VPS35, Retinal ganglion cell, Neuro-degeneration, Optic nerve injury

## Abstract

VPS35 (vacuolar protein sorting 35) is a major component of retromer that selectively promotes endosome-to-Golgi retrieval of transmembrane proteins. Dysfunction of retromer is a risk factor for the pathogenesis of Parkinson’s disease (PD) and Alzheimer’s disease (AD), both neuro-degeneration disorders. However, VPS35/retromer’s function in retina or the contribution of Vps35-deficiency to retinal neuro-degenerative disorders has not been investigated. Here we provide evidence for a role of VPS35 in mouse retinal ganglion cell (RGC) survival and regeneration. VPS35 is selectively expressed in developing mouse RGCs. RGCs from young adult Vps35 heterozygotes (Vps35^+/m^) show degenerative-like features, such as dystrophic dendrites, reduced axon fibers, and increased TUNEL labeled RGCs. Additionally, gliosis in the optic nerve is transiently elevated in neonatal, but reduced in aged Vps35^+/m^ mice. Optic nerve injury-induced gliosis is also attenuated in Vps35^+/m^ mice. These results suggest that Vps35 is necessary for mouse RGC survival and regeneration, and Vps35-deficiency may contribute to the pathogenesis of retinal ganglion neuro-degeneration, a critical pathology leading to the blindness of many retinal degenerative disorders.

## Background

Retromer is essential for selective retrieval of transmembrane proteins from endosomes to *trans-Golgi* network [1-3]. Retromer contains two sub-protein complexes: the cargo-selective complex and membrane deformation complex [2,4]. VPS35 is the key component of the cargo-selective complex, a trimer of VPS proteins VPS35, VPS29, and VPS26. Dysfunction of VPS35/retromer is a risk factor for neuro-degenerative disorders, including Parkison’s disease (PD) and Alzheimer’s disease (AD) [5-9]. Mutations in Vps35 gene is identified in patients of late-onset PD [5,6]. The retromer complex (e.g., Vps35 and Vps26) is decreased in the postmortem hippocampus of AD patients [7]. In Vps35 or Vps26 deficient animals, the major culprit of AD, β-amyloid (Aβ), is increased in the hippocampus [7,9]. In addition, Vps35 haploinsufficiency in Tg2576 mouse model of AD enhances Aβ-associated neuropathology [9]. Furthermore, using in utero electroporation, suppression of Vps35 expression in embryonic hippocampal neurons results in “degenerative-like” phenotypes [10]. These observations thus suggest a critical role for VPS35/retromer in preventing neuro-degeneration.

Retinal ganglion cells (RGCs), important neurons in the retina, receive visual information from photoreceptors via two intermediate neurons (bipolar and amacrine cells) and deliver the signal via their axons. RGC’s axons form nerve fibers, extend into the optic nerve through optic disc, further form the optic chiasm and optic tract, and finally transmit visual information from the eye to the brain. Dysfunction of RGCs or degeneration of RGCs is a major pathology detected in several retinal disorders, including glaucoma [11,12] and age-related macular degeneration(AMD) [12,13], which is a main reason leading to irreversible blindness.

Although dysfunction of Vps35 is implicated in the pathogenesis of AD and PD, its function in the retina is largely unknown. We thus asked: where in the retina Vps35 is expressed? Does loss of Vps35 contribute to the retinal neuron degeneration? Here, we started to shed light on these questions by demonstrating Vps35 expression selectively in mouse RGCs. RGCs in *Vps35* heterozygote mice (named vps35^+/m^) exhibit degenerative-like morphology, such as disturbed dendritic processes, reduced axonal fibers, and increased RGC apoptosis. In addition, Vps35^+/m^ mice show impairment in optic nerve injury-induced gliosis, implicating Vps35 in regulating optic nerve regeneration.

## Results

### VPS35 expression in developing mouse RGCs, including melanopsin positive ipRGCs

**Figure 1:**
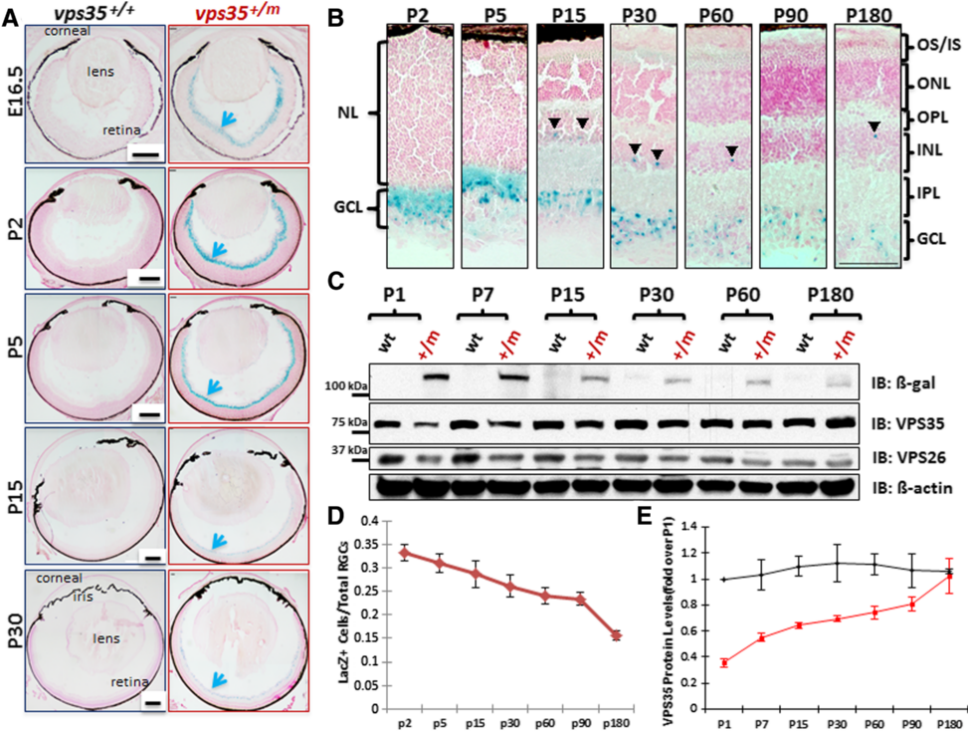
**Vps35 expression in developing mouse retinas. (A)** X-gal staining (Blue color) showed *lacZ gene* expression in the retina of vps35^+/m^ mice at indicated ages. Scale bar, 200 μm. **(B)** The high magnification of X-gal staining images was shown. GCL: ganglion cell layer; IPL: inner plexiform layer; INL: inner nuclear layer; OPL: outer plexiform layer; ONL: outer nuclear layer; OS: outer segment; IS, inner segment. Scale bar, 50 μm. In addition to GCL, a few LacZ positive cells were detected in INL indicated by arrow heads. **(C)** Western blot analysis of VPS35 expression in retina homogenates from vps35^+/+^ and ^+/m^ at indicated ages. **(D)** Quantification analysis of percentage of LacZ positive cells. **(E)** Quantification analysis of VPS35 protein levels in retina from indicated age-groups of vps35^+/+^ and ^+/m^ mice. In (D-E), mean+/-SEM (n = 5) were presented.

To investigate the potential role of VPS35 in retina, we first examined vps35’s expression in mouse retina by taking advantage of vps35^+/m^ mice, in which LacZ gene is knocked in the intron of vps35 gene, thus, the LacZ activity, under the control of vps35 promoter, can be used as a reporter for vps35’s expression [9,10,14]. The *LacZ activity* was detected in developing mouse retinas from embryonic 12.5 to all the ages examined (Figure 1A and data not shown). Interestingly, this LacZ activity was mainly distributed in the ganglion cell layer (GCL) of vps35^+/m^ retinas (Figure 1A). Higher power imaging analysis showed few X-gal (ß-galactosidase) positive cells in the inner plexiform (IPL) and upper inner nuclear (INL) layers, in addition to GCL (Figure 1B). Note that not all of the cells in GCL were LacZ positive (Figure 1B), and the ratio of *LacZ* positive cells over total cells in GCL was around 30%, which was reduced in aged retina (Figures 1A-D). These results suggest that vps35 is mainly expressed in developing mouse RGCs, and may be decreased during aging.

We then examined VPS35’s expression by Western blot analysis. Consistently, An age-dependent reduction of ß-galactosidase protein was observed in vps35^+/m^ retinas (Figures 1C-D). However, using anti-VPS35 antibody, the VPS35 protein levels were comparable among different aged vps35^+/+^ retinas (Figures 1C and E). ~50% reductions of Vps35 proteins were detected in young (P1 to P60), but not aged (P90 to P180), Vps35^+/m^ retinas (Figures 1C and E), implicating a compensatory effect in aged mutant mice. The differential results by anti-ß-galactosidase and anti-VPS35 antibodies may result from an age-dependent down-regulation of vps35’s transcriptional expression (viewed by its LacZ reporter) or ß-galactosidase protein stability, but not VPS35 protein. To distinguish these possibilities, we examined vps35’s transcripts from different aged Vps35^+/+^ and ^+/m^ retinas by real time PCR (RT-PCR) analysis. Vps35’s mRNAs appeared to be unchanged among various aged Vps35^+/+^ retinas, but decreased in Vps35^+/m^ controls (Additional file 1: Figure S1A). This result demonstrates the specificity of the analysis and excludes the possibility for age-dependent inhibition of vps35’s transcription. In line with this view, little change was observed for the LacZ transcripts detected in various aged Vps35^+/m^ retinas, but not in Vps35^+/+^ controls (Additional file 1: Figure S1B). Taken together, these results suggest that Vps35 appears to be stably expressed in mouse retina throughout development, and the reduced LacZ staining/protein level in aged mutant retina may be due to an-age dependent instability of the LacZ protein. The stable VPS35 expression in different aged retina GCL was further supported by immunostaining analysis (Additional file 1: Figure S1C). Note that vps35^+/m^ retina (particularly at neonatal age) showed ~50% reduction of VPS35 protein (Figures 1C, E), demonstrating the antibody specificity. VPS26, another component of retromer, was also decreased in vps35^+/m^ retina (Figure 1C). These results thus suggested that the retromer complex is stably expressed in developing mouse retina, which is mainly distributed in the retinal GCL and lost in vps35^+/m^ mice.

**Figure 2:**
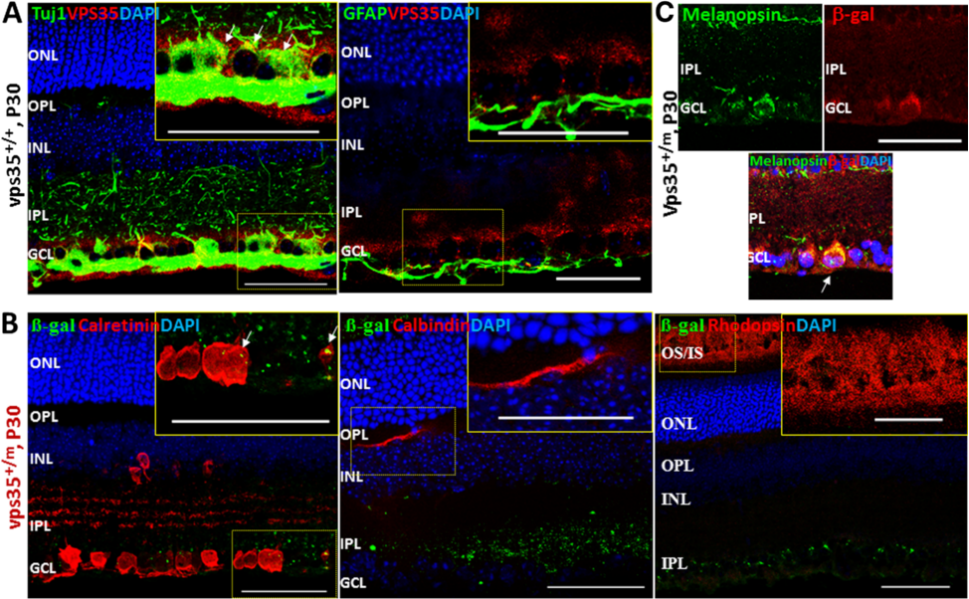
**VPS35 expression in mouse RGCs.** Co-immunostaining analysis was carried out in cross sections from P30 Vps35^+/+^**(A)** and ^+/m^**(B-C)** mouse retinas using indicated antibodies. GCL, ganglion cell layer; NL, neuroblast layer; IPL, inner plexiform layer; INL, inner nuclear layer; OPL, outer plexiform layer; ONL, outer nuclear layer; OS, outer segment; IS, inner segment. Arrows indicate co-localization signals. Inserts are amplified images of the marked squares. Scale bar, 50 μm.

Retinal ganglion cells (RGCs) are largely distributed in the GCL. We thus examined if VPS35 is expressed in RGCs by co-immunofluorescence staining analysis using anti-VPS35 or anti-β-gal antibodies in vps35^+/+^ or ^+/m^ retinas. As shown in Figure 2A, VPS35 was largely co-distributed with Tuj1, which recognizes neuronal class β-III tubulin in RGCs. It was undetectable in horizontal cells (marked by calbindin) and photoreceptor cells (by rhodopsin), but weakly detected in amacrine cells (viewed by calretinin) and glial cells (by GFAP) (Figures 2A-B). These results thus provide evidence for the selective expression of VPS35 in mouse RGCs.

Note that a few β-gal positive cells were also distributed in IPL (inner plexiform layer), in addition to GCL layer (Figure 1B). Such a distribution pattern is similar to that of M1 type of ipRGCs (intrinsically photo-sensitive retinal ganglion cells) that express melanopsin [15,16]. We thus examined if Vps35 is co-distributed with melanopsin in ipRGCs. Indeed, fractions, but not all, of β-Gal positive cells were also melanopsin positive (Figure 2C). Taken together, these results suggested that VPS35 is expressed in developing mouse RGCs, including ipRGCs.

### Degenerative-like deficit in vps35^+/m^ RGCs

Next we asked whether VPS35 plays a role in RGC development and survival. RGC starts to develop around E10.5 and finished its development in neonatal age [17]. We carried out immunostaining analysis of P1 flat-mounted eye disc using antibodies against neurofilament or Tuj1, which recognize RGCs’ soma, axons, and dendrites (by Tuj1). A normal distributed RGC neuronal somas and axonal fibers in Vps35^+/m^ retina were observed (Additional file 2: Figure S2), suggesting little, if there is any, role of VPS35 in RGC development.

**Figure 3:**
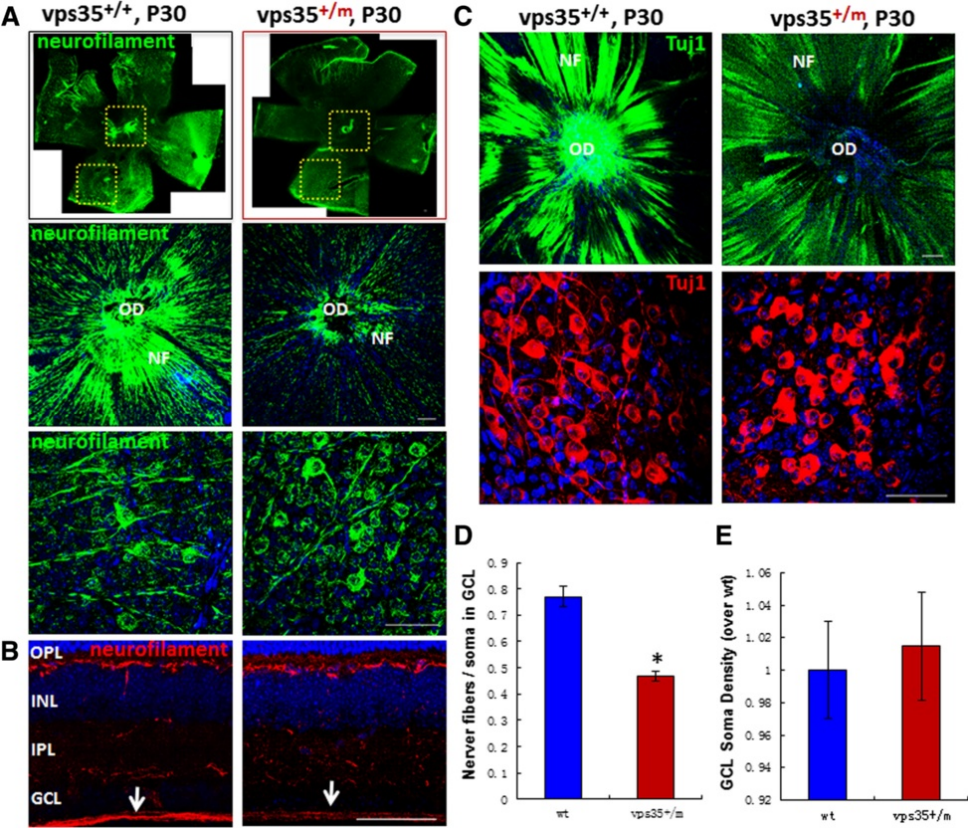
**Reduced RGC axon fibers in P30 vps35**^**+/m **^**retinas. (A-B)** Immunostaining analysis using anti-neurofilament antibodies of flat-mounted retinas **(A)** and cross sections **(B)** from P30 Vps35^+/+^ and ^+/m^ mice. In A, the bottom two panels are amplified images from the marked squares in upper panel. OD: optic disc; NF: nerve fiber. **(C)** Immunostaining analysis using anti-Tuj1antibodies of flat-mounted retinas from P30 Vps35^+/+^ and ^+/m^ mice. Scale bars in A-C, 50 μm. **(D)** Quantification analysis of the ratio of nerve fibers verse RGC somas stained by anti-Tuj1. **(E)** Quantification analysis of RGC soma density. In (D-E), mean +/- SEM (n = 5 retinas) were shown. *, P < 0.05.

We then examined RGCs in both flat-mounted and cross-sectioned retina derived from P30 vps35^+/+^ and ^+/m^ mice by immunostaining analysis of neurofilament. Whereas the numbers and distribution of RGC somas appeared to be normal in vps35^+/m^ retina, RGC nerve fibers labeled by neurofilament were reduced in vps35^+/m^ retinas as compared to that in vps35^+/+^ controls (Figures 3A-E), implicating axonal deficit of RGCs in vps35 mutant retina. Being consistent, immunostaining analysis using anti-Tuj1 antibody also showed reduced Tuj1^+^ RGC axons, but not somas, in the mutant retinas (Figures 3C-E). These alterations in vps35^+/-^ retina resembled to that of dystrophic RGC axons.

**Figure 4:**
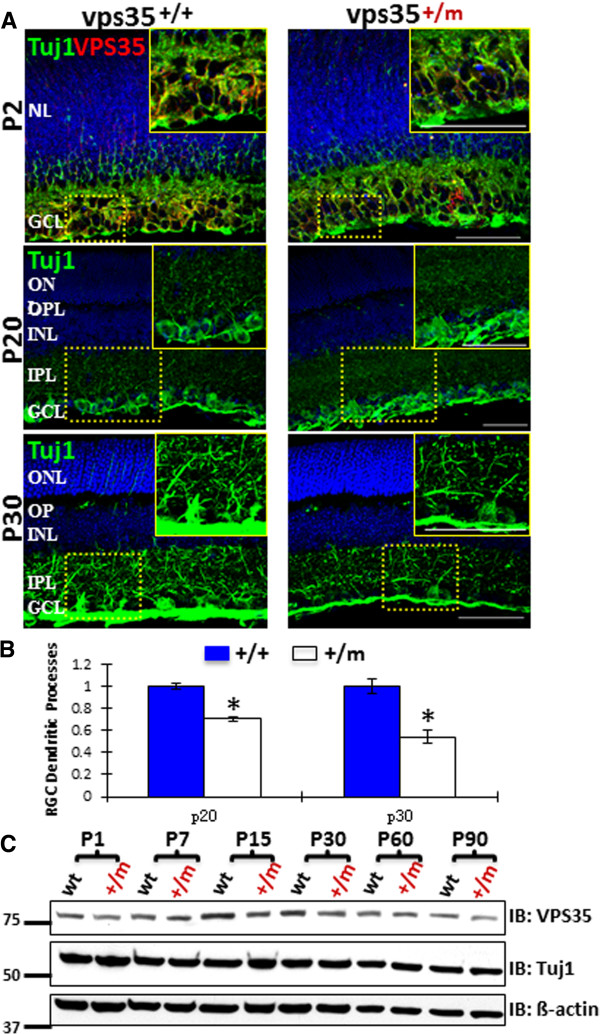
**Disturbed RGC dendrites in P20 vps35**^**+/m **^**retinas. (A)** Immunostaining analysis using anti-Tuj1 antibodies of cross-sections of retinas from vps35^+/+^ and ^+/m^ mice at indicated ages. Inserts are amplified images of the marked squears. GCL, ganglion cell layer; NL, neuroblast layer; IPL, inner plexiform layer; INL, inner nuclear layer; OPL, outer plexiform layer; ONL, outer nuclear layer; OS, outer segment; IS, inner segment. Arrows indicate co-localization signals. Inserts are amplified images of the marked squares. Scale bar, 50 μm. **(B)** Quantification analysis of the fluorescence intensity in RGC dendrites marked by anti-Tuj1. The values of mean +/- SEM (n = 5 retinas) were shown. *, P < 0.05. **(C)** Western blot analysis of Tuj1 expression in retina homogenates from vps35^+/+^ and ^+/m^ at indicated ages.

We further examined RGC dendritites in cross-sectioned retina from P30 vps35^+/+^ and ^+/m^ mice. Tuj1-labeled RGC dendritic processes appeared to be normal in P2 vps35^+/m^ retina (Figure 4A). At age of P20, RGC dendrites were obviously disturbed in the IPL of vps35^+/m^ retina with a reduced Tuj1 staining signal (Figures 4A-B). However, western blot analysis using whole retina homogenates showed no obvious difference in Tuj1 protein levels between vps35^+/+^ and ^+/m^ mice (Figure 4C). This may be due to the large amounts of Tuj1 proteins distributed in the soma and the soma density unchanged or slightly increased in the mutant as compared to that of wild type controls (Figures 3C-E). The over-all retina morphology, revealed by H & E staining analysis, appeared to be normal in vps35^+/m^ retina as compared to that of vps35^+/+^ controls (Additional file 3: Figure S3). Taken together, these results revealed axonal and dendritic degenerative-like deficits in RGCs, but not in other neurons, in vps35 mutant retina.

**Figure 5:**
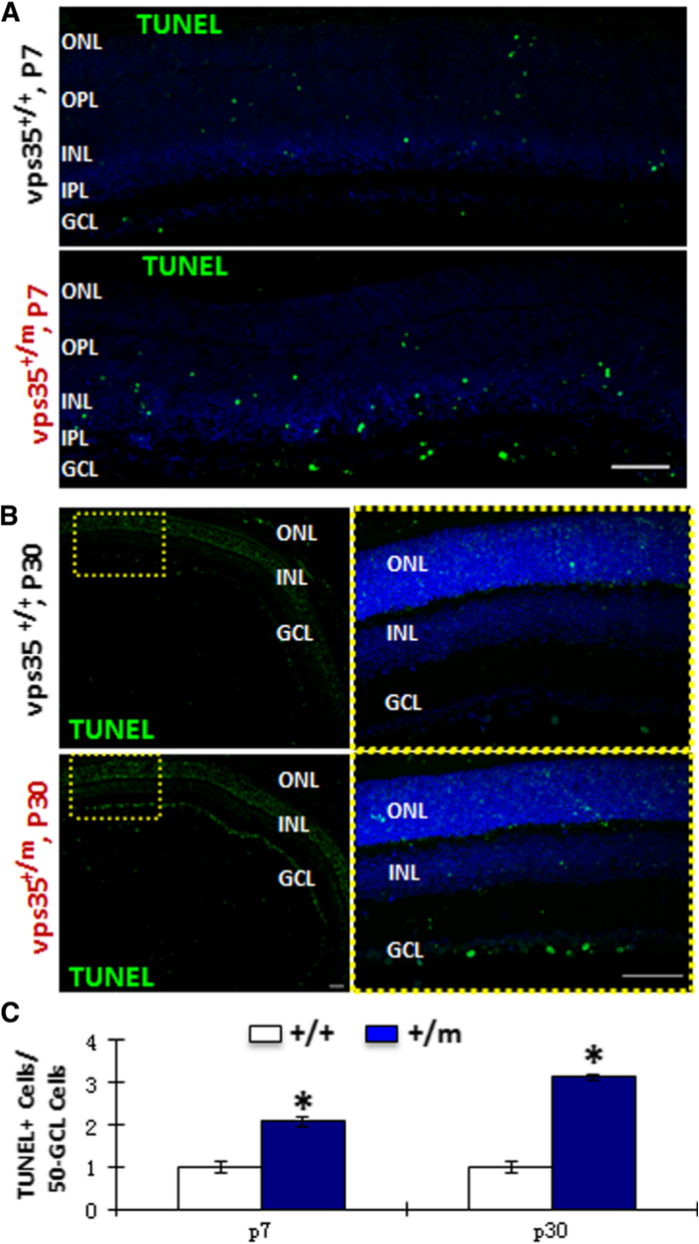
**Increased TUNEL positive RGCs in vps35**^**+/m **^**retinas.** TUNEL staining analysis was carried out in cross-sections of retinas from P7 **(A)** and P30 **(B)** vps35^+/+^ and ^+/m^ mice . GCL, ganglion cell layer; IPL, inner plexiform layer; INL, inner nuclear layer; OPL, outer plexiform layer; ONL, outer nuclear layer. Scale bar, 50 μm. The TUNEL^+^ cells in GCL were quantified and showed as mean +/- SEM (n = 3) in **(C)**. *, P < 0.05.

We then asked if VPS35 plays a role in mouse RGC survival. TUNEL assay, which label DNA damaged cells, was carried out in vps35^+/+^ and ^+/m^ retinas. Remarkably, increased TUNEL positive cells, particularly in the RGC layer of both P7 and P30 vps35^+/m^ retina, were detected as compared to that of same age groups of vps35^+/+^ controls (Figure 5). Thus, these results support the view for a critical role of VPS35 in suppressing developing RGC degeneration.

### Reduced optic nerve injury-induced gliosis in neonatal Vps35^+/m^ mice

**Figure 6:**
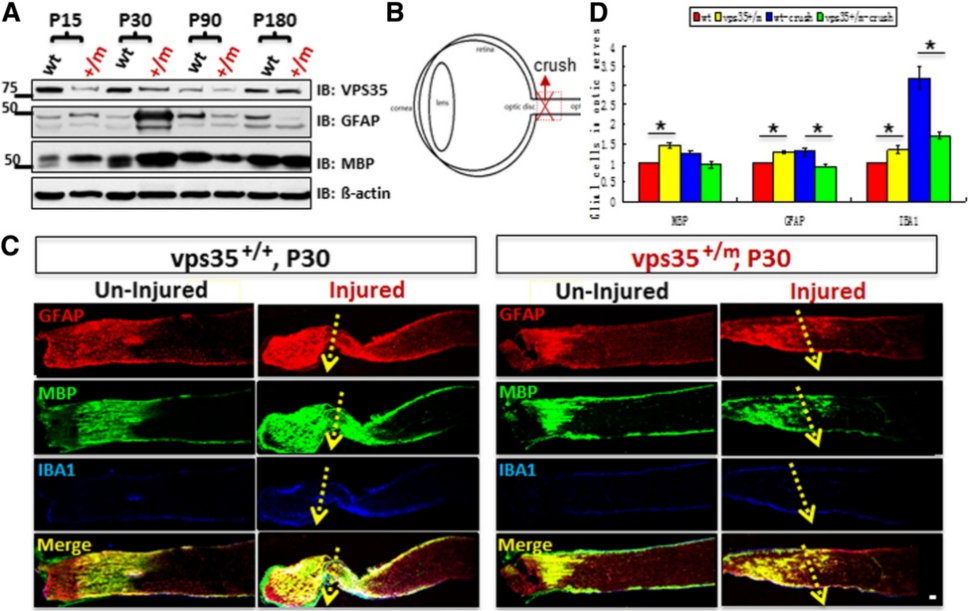
**Impairment of optic nerve injury-induced gliosis in vps35**^**+/m **^**mice. (A)** Western blot analysis of homogenates from vps35^+/+^ and ^+/m^ optic nerves at indicated ages using indicated antibodies. **(B)** Illustration of mouse optic nerve injury. P30 vps35^+/+^ and ^+/m^ optic nerves were crushed as indicated. 3-days after injury, optic nerves were sectioned and subjected to immunostaining analysis. **(C)** Immunostaining analysis of optic nerves using indicated antibodies. Injury sites were marked with yellow arrows. Scale bar, 50 μm. **(D)** Quantification analysis of data from **(C)**. Immunofluorescence intensity was normalized by the uninjured vps35^+/+^ controls. The values of mean +/- SEM (n = 3) were shown. *, P < 0.05, significance difference from the vps35^+/+^ control.

Optic nerves are assembled by RGC axonal fibers and glial cells, such as oligodendricytes, astrocytes, and microglia [18]. To examine whether optic nerves are altered in vps35^+/m^ mice, we first examined Vps35’s expression in optic nerve homogenates from various aged vps35^+/+^ and ^+/m^ mice. Vps35 protein levels were high in neonatal age (P15 and P30), but reduced in aged vps35^+/+^ mice (Figure 6A), in contrast from that in mouse retinas (Figure 1C). In agreement with the data from aged retinas, Vps35 protein levels were only decreased to ~50% in young age (P15 to P90), but not aged (e.g., P180), optic nerves (Figure 6A). Interestingly, increased GFAP (a marker for astrocytes) and MBP (myelin binding protein which marks oligodendricytes) levels were increased in neonatal, but decreased in aged (e.g., P90 and P180), vps35^+/m^ optic nerves (Figure 6A). We thus further examined optic nerve phenotype in P30 vps35^+/+^ and ^+/m^ mice by immunostaining analysis using antibodies against Tuj1 (to label RGC axons), GFAP (to mark astrocytes), MBP (to view oligodendricytes), and IBA-1 (to stain microglia). RGC axons didn’t show obvious difference between P30 vps35^+/+^ and ^+/m^ optic nerves, based on immunostaining and Western blot analyses using anti-Tuj1 antibodies (data not shown). Oligodendricytes labeled by anti-MBP antibody were absent in the retina and optic nerve head, but present posterior to the lamina cribrosa-like region in both vps35^+/+^ and ^+/m^ optic nerves (Figure 6C). In contrast, astrocytes marked by GFAP were present in the retina and optic nerve head (Figure 6C). In agreement with data from Western blot analysis, Glial cells labeled by GFAP, MBP, and IBA-1 antibodies were all slightly increased in P30 vps35^+/m^ optic nerves (Figures 6C-D). These results thus suggest an age-dependent alteration of gliosis in vps35^+/m^ mutant optic nerves.

We then asked if Vps35 deficiency affects optic nerve injury-induce gliosis, a critical process associated with optic nerve regeneration [19]. Optic nerves were crushed at the site close to the optic disc (Figure 6B). 3-days after injury, mice were sacrificed and optic nerves were examined by immunostaining analysis. Upon nerve injury, gliosis, viewed by anti-GFAP, MBP, and IBA1 antibodies, was increased in vps35^+/+^ optic nerves (Figures 6C-D). MBP-labeled oligodendritecytes appeared to be shifted to the site of optic disc (Figures 6C-D). In contrast, the nerve injury induced gliosis, marked by GFAP and IBA1 antibodies, was attenuated in vps35^+/m^ mice (Figures 6C-D), suggesting an impairment of optic nerve injury-induced gliosis.

## Discussion and conclusions

VPS35 is a key element of retromer that is a heteropentameric complex with a transmembrane protein sorting function from the endosome to the *trans-*Golgi network [1-3]. Dysfunction of VPS35/retromer is a risk factor for a number of neurodegenerative disorders, including PD and AD [5-9,20]. Given VPS35’s crucial function in suppressing neuro-degeneration, we asked if it plays a role in retinal neuron survival. Here, we presented several lines of evidences for a critical role of VPS35 in mouse RGC survival. First, VPS35 is selectively expressed in developing mouse RGCs. Second, vps35^+/m^ retina show degenerative-like RGCs, with disturbed RGC dendrites, reduced RGC axon fibers, and increased apoptosis. Third, vps35^+/m^ optic nerves exhibit transient elevation of gliosis in neonatal age, but reduced in aged mice. Finally, optic nerve injury-induced gliosis is attenuated in vps35^+/m^ mice.

Several characteristics of Vps35’s expression in mouse retina are noted. It is selectively expressed in mouse RGCs. This is based on in large the distribution of the LacZ reporter in the Vps35 gene. This view is also confirmed by immunostaining analysis using anti-VPS35 antibody. The LacZ reporter expression in RGCs is reduced during aging. However, no obvious reduction in Vps35 protein levels was detected by Western blot analysis of the retina homogenates. These results implicate that Vps35’s protein may be very stable, but its transcript could be reduced during aging. In addition, VPS35 is expressed in subset of RGCs, including ipRGCs. However, the function of VPS35 in ipRGCs remains to be further investigated.

The degenerative-like morphology of vps35^+/-^ RGCs may be largely caused by the reduced VPS35 expression in RGCs. This view is in line with the observation of the selective expression of VPS35 in developing RGCs. However, we cannot exclude the possibility of cell non-autonomous mechanisms, as Vps35 is also weakly expressed in glial cells, such as astrocytes and microglia, and vps35^+/m^ mice show altered gliosis (Figure 6) and increased inflammation [14]. The latter may contribute further RGC degeneration.

Gliosis, a nonspecific reactive change of glial cells in response to damage to the neurons, involves the proliferation of different types of glial cells, including astrocytes, microglia, and oligodendrocytes. Gliosis has both beneficial and detrimental effects. Its beneficial effects include neuroprotection, maintenance of the extracellular environment, seclusion of the injury site, and nerve regeneration [21,22]. Its detrimental effects are restriction of axon regeneration when there is glial scar formation. Vps35^+/m^ mice showed an-age dependent gliosis, detected in neonatal, but not aged, mice. Such a transient gliosis may be the consequences of the RGC degeneration. Interestingly, optic nerve injury-induced gliosis was attenuated in Vps35^+/m^ mice, implicating that VPS35 may play a role in nerve regeneration.

The retinal degeneration is a major pathology associated with glaucoma [11,12] and age-related macular degeneration (AMD) [12,13]. It is of interest to note that glaucoma or AMD is often associated with other neurodegenerative disorders, including AD and PD [12]. Mutations in Vps35 gene are identified in late onset PD patients [5,6]. Dysfunction of retromer is also believed to be a risk factor for AD [7,9]. However, it remains unknown if there is any mutation in vps35 gene or other retromer component in patients with glaucoma or AMD. Given the fact we demonstrate here that VPS35 regulates RGC survival, plus the association of retinal RGC degeneration with neuro-degeneration, it is conceivable that dysfunction of VPS35 or other retromer component may contribute to the pathogenesis of glaucoma or AMD patients. Clearly, this requires further analysis of the putative causative genes in glaucoma or AMD patients.

## Methods

### Animals and reagents

Vps35 mutant mice have been described previously [9,10,14], which were backcrossed with C57BL/6 mice for more than 10 generations. Mice were maintained on a standard rodent diet. Vps35 mutations were confirmed by genotyping using PCR and Western blot analysis. All experimental procedures were approved by the Animal Subjects Committee at the Georgia Regents University according to US National Institutes of Health guidelines.

Rabbit polyclonal anti-VPS35 antibody was generated using the antigen of GST-VPS35D1 fusion protein as described previously [9,10,14]. Rabbit polyclonal antibodies, including anti- ß-galactosidase (Cappel), anti-VPS26 (Abcam),anti-calretinin (Swant), and anti-melanopsin (Advanced Targeting Systems) antibodies, were purchased. Mouse monoclonal antibodies, including anti-neuronal class III ß-Tubulin (Tuj1,Convance), anti-neurofilament (DSHB), anti-calbindin D28k (Swant), anti-glial fibrillary acidic protein (GFAP, Chemicon), anti-rhodopsin (Abcam) and anti-myelin basic protein (MBP, Chemicon) antibodies, were also purchased. In addition, the chicken polyclonal anti- ß-galactosidase antibody (Abcam) and goat polyclonal anti-IBA1 antibody (Abcam) were used. Secondary antibodies were purchased from Jackson Immuno Research Laboratories, Inc. Other chemicals and reagents used in this study were of analytical grade.

### β-Gal detection, immunofluorescence staining, and confocal imaging analysis

Vps35^+/+^ or ^+/m^ mice were sacrificed and the eyes were excised. For β-Gal detection, the eyes were immediately embedded in OCT medium (Sakura Finetek, Torrance, CA), and frozen at -80°C for 2h. 20 μm-thick sections were cut on a cryostat and mounted onto Super-Frost Plus slides. For immunofluorescence staining, after eyes excised, the corneas were removed, eye cups were fixed in 4% paraformaldehyde in 0.1 M PBS at 4°C for 24 h. Lenses were then removed, the retina to be flat-mounted were dissected from eye cups and processed in 1.5ml microfuge tubes. Eye cups for cross sectioning were cryo-protected with 30% sucrose in PBS at 4°C for 24 h, embedded in OCT medium (Sakura Finetek, Torrance, CA), frozen, sectioned at 12 μm-thickness, and mounted onto Super-Frost Plus slides.

β-Gal activity was detected as described previously [9,10,14,23,24]. In brief, frozen eye sections derived from Vps35^+/+^ and Vps35^+/m^ mice were fixed with 0.5% glutaraldehyde and incubated with X-gal solution (2 mM MgCl2, 5 mM potassium ferricyanide, 5 mM potassium ferrocyanide, and 0.1% X-gal) in the dark at 37°C for 12 h. The slides were washed, mounted in permount (Thermo Fisher Scientific), and imaged using deconvolution digital microscope (Axioplan 2; Carl Zeiss) with high sensitivity camera (AxioCam; Carl Zeiss) and equipped with a plan-neofluar 5× and plan-neofluar 10×/0.30 NA objective lens.

For immunofluorescence staining analysis, flat mounts or cross-sections were post-fixed with 4% PFA at room temperature for 15 min, permeabilized with 0.3% Triton X-100 at room temperature for 20 min, and blocked with 10% horse serum and 5% BSA at room temperature for 2 h. Flat mounts or sections were incubated for 24 h at 4°C in primary antibodies. Antibodies used were rabbit polyclonal anti-VPS35 (1:1,000), mouse monoclonal anti-Tuj1 (Convance, 1:1,000), mouse monoclonal anti-neurofilament (DSHB, 1:1,000), rabbit polyclonal anti-calretinin (Swant, 1:500), rabbit polyclonal anti-melanopsin (Advanced Targeting Systems, 1:5,000), mouse monoclonal anti-calbindin D28k (Swant, 1:2,000), mouse monoclonal anti-GFAP (Chemicon, 1:1,000), mouse monoclonal anti-Rhodopsin (Abcam, 1:500), mouse monoclonal anti-MBP (Chemicon, 1:500), goat polyclonal anti-IBA1 antibody (Abcam, 1:500) and chicken polyclonal anti-ß-galactosidase (Abcam, 1:1,000). Flat mounts or sections were incubated with appropriate fluorescent secondary antibodies at room temperature for 2 h, then incubated with Topro3 (1:5,000) at room temperature for 10 min. Slides were mounted using VECTASHIELD mounting medium. Confocal images were obtained using Nikon C1 confocal system. For fluorescent quantification, morphometric measurements of images were performed using Image- pro plus software (Media Cybernetics).

### Tissue lysis and western blotting

Retina and optic nerve tissues were lysed in lysis buffer (50 mM Tris-HCl (pH 7.4), 150 mM NaCl, 1% NP-40, 0.5% Triton X-100, 1 mM phenylmethylsulfonyl fluoride (PMSF), 1 mM EDTA, 5 mM sodium fluoride, 2 mM sodium orthovanadate and protease inhibitors) 15 minutes at 4°C and centrifuged at 14,000 × g, protein concentration was determined by BCA protein assay kit (Thermo). Resulting supernatants separated by SDS-PAGE in 8%–12% gradient gels. Electroblotting was at 4°C for 2 hours (100V, 400 mA). Membranes were blocked in 10% silk milk at room temperature for 1h. Primary antibody incubation was overnight at 4°C. After 3× washes with TBST (Tris-Buffered Saline and Tween 20), membranes were incubated in secondary antibodies for 1 h at room temperature. Primary antibodies included rabbit polyclonal anti-VPS35 (1:10,000), rabbit polyclonal anti-VPS26 (Abcam, 1:2,000), rabbit polyclonal anti-ß-galactosidase (Cappel, 1:2,000), mouse monoclonal anti-Tuj1 (Convance, 1:4,000), mouse monoclonal anti-GFAP (Chemicon, 1:1,000) and mouse monoclonal anti-MBP (Chemicon, 1:1,000). Secondary antibodies (all 1:8,000 dilution; all Thermo) were anti-mouse and anti-rabbit HRP. Band intensities were quantified densitometrically and normalized to ß-actin. For semi-quantitative analysis, protein bands detected by ECL were scanned into Adobe Photoshop CS5 and analyzed using ImageJ software (National Institutes of Health). Care was taken during exposure of the ECL film to ensure that intensity readouts were in a linear range of standard curve blot detected by the same antibody.

### Terminal deoxynucleotidyl transferase-mediated biotinylated UTP nick end labeling (TUNEL)

Sections were processed for TUNEL as described above. TUNEL assay was performed using the In Situ Cell Death Detection Kit (Roche).

### Optic nerve injury

Optic nerve injury was performed in Vps35^+/+^ and Vps35 ^+/m^ mice at P30 as described previously [19]. Only the left optic nerve was injured, the right optic nerve served as negative control. Mice were anesthetized by intraperitoneal injection of Ketamine and Xylazine (100 mg/kg and 20 mg/kg body weight). The incision was made in the conjunctiva at the superior pole of the eye, then a blunt dissection of the conjunctiva was made with forceps towards the back of the eye to expose the retrobulbar optic nerve. The optic nerve was crushed with forcep for 10 seconds at 1 mm behind the globe. After the surgery, tobramyclin ointment was applied to avoid infection and the animal recovered on a warming pad. After 3 days, mice were sacrificed, the eyes were excised, then removed the optic nerves from the eye-balls. The optic nerves were fixed in 4% paraformaldehyde in 0.1 M PBS at 4°C for 24 h and cryo-protected with 30% sucrose in PBS at 4°C for 24 h, embedded in OCT medium(Sakura Finetek, Torrance, CA), frozen, sectioned 12 μm-thickness, and mounted onto Super-Frost Plus slides.

### Statistical analysis

All data were expressed as means ± SEM. Three to five mice per genotype per assay were used. Three to five positions were quantified for immunostaining analyses. The significance level was set at P < 0.05, and the Student’s t test was used.

## Competing interests

All authors have no conflicts of interest.

## Authors’ contributions

WL carried out most of the experiments and involved in data analysis and manuscript writing. FLT maintained mutant mice and helped on all of the experimental design and data analysis. JE performed the experiments of real time PCR analysis and involved in data analysis. HX provided reagents and advice on some of the experiments. JY provided advice on experimental design, data analysis, and manuscript writing. WCX involved in experimental design, data analysis, and manuscript writing. All authors read and approved the final manuscript.

## Supplementary Material

Additional file 1: Figure S1 Vps35 expression in various aged mouse retinas. **(A)** Real time PCR analysis showed *vps35’s* expression in vps35^+/+^ mouse retinas at indicated ages, which was reduced in the same age-groups of vps35^+/m^ retinas. **(B)** Real time PCR analysis showed *LacZ* transcripts only in vps35^+/m^, but not in vps35^+/+^, mouse retinas at indicated ages, suggesting the specificity of the RT-PCR analysis. In (A-B), mean +/- SEM (n = 3) were shown. **(C)** Immunostaining analysis using anti-VPS35 antibody showed Vps35’s distribution in GCL of Vps35^+/+^ mouse retina at indicated ages, which was also reduced in Vps35^+/m^ retinas, demonstrating the antibody specificity. Scale bar, 100 μm. Click here for file

Additional file 2: Figure S2 RGC axon fibers in P1 vps35^+/m^ retinas. **(A)** Immunostaining analysis using anti-neurofilament antibodies of flat-mounted retinas from P1 Vps35^+/+^ and ^+/m^ mice. OD: optic disc; NF: nerve fiber. Scale bars, 50 μm. **(B)** Quantification analysis of the ratio of nerve fibers verse RGC somas stained by anti-neurofilament. The mean +/- SEM ( n = 3) were presented. Click here for file

Additional file 3: Figure S3 H & E staining analysis of retina morphology in vps35^+/+^ and ^+/m^ mice at indicated ages. GCL, ganglion cell layer; NL, neuroblast layer; IPL, inner plexiform layer; INL, inner nuclear layer; OPL, outer plexiform layer; ONL, outer nuclear layer; OS, outer segment; IS, inner segment. Scale bar, 50 μm. Click here for file
