# Supplementary figures and images for: Vps35 haploinsufficiency results in degenerative-like deficit in mouse retinal ganglion neurons and impairment of optic nerve injury-induced gliosis
Source: Mol Brain. 2014 Feb 11;7:10. doi: 10.1186/1756-6606-7-10 (PMC4016418; doi:10.1186/1756-6606-7-10)

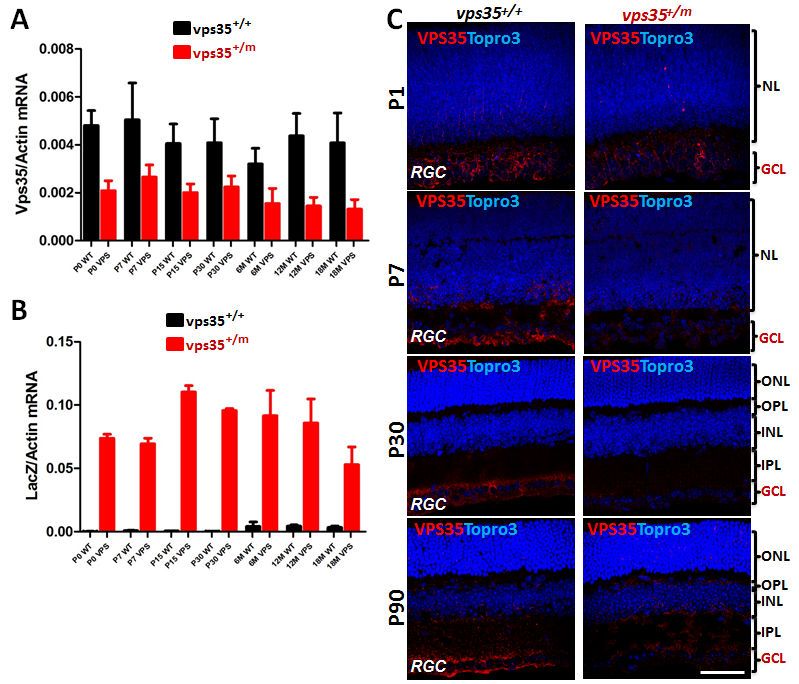

Supplement: Additional file 1: Figure S1 — Vps35 expression in various aged mouse retinas. (A) Real time PCR analysis showed vps35’s expression in vps35+/+ mouse retinas at indicated ages, which was reduced in the same age-groups of vps35+/m retinas. (B) Real time PCR analysis showed LacZ transcripts only in vps35+/m, but not in vps35+/+, mouse retinas at indicated ages, suggesting the specificity of the RT-PCR analysis. In (A-B), mean +/- SEM (n = 3) were shown. (C) Immunostaining analysis using anti-VPS35 antibody showed Vps35’s distribution in GCL of Vps35+/+ mouse retina at indicated ages, which was also reduced in Vps35+/m retinas, demonstrating the antibody specificity. Scale bar, 100 μm. [file 1756-6606-7-10-S1.tiff]

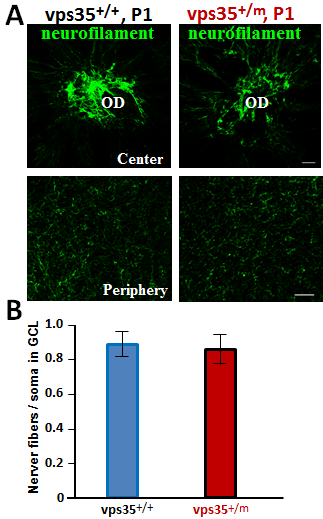

Supplement: Additional file 2: Figure S2 — RGC axon fibers in P1 vps35+/m retinas. (A) Immunostaining analysis using anti-neurofilament antibodies of flat-mounted retinas from P1 Vps35+/+ and +/m mice. OD: optic disc; NF: nerve fiber. Scale bars, 50 μm. (B) Quantification analysis of the ratio of nerve fibers verse RGC somas stained by anti-neurofilament. The mean +/- SEM ( n = 3) were presented. [file 1756-6606-7-10-S2.tiff]

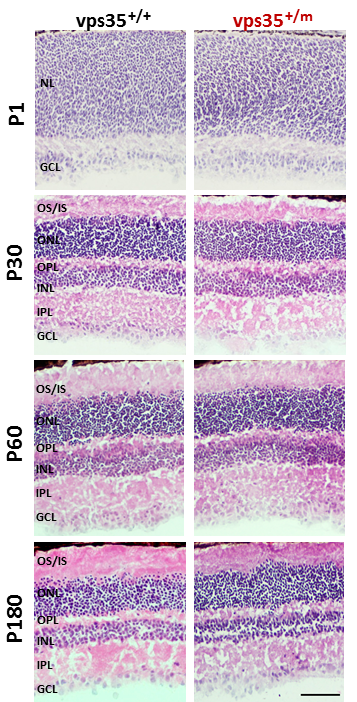

Supplement: Additional file 3: Figure S3 — H & E staining analysis of retina morphology in vps35+/+ and +/m mice at indicated ages. GCL, ganglion cell layer; NL, neuroblast layer; IPL, inner plexiform layer; INL, inner nuclear layer; OPL, outer plexiform layer; ONL, outer nuclear layer; OS, outer segment; IS, inner segment. Scale bar, 50 μm. [file 1756-6606-7-10-S3.tiff]
